# Tunable Bandgap and Optical Properties of Black Phosphorene Nanotubes

**DOI:** 10.3390/ma11020304

**Published:** 2018-02-19

**Authors:** Chunmei Li, Zhongjing Xie, Zhiqian Chen, Nanpu Cheng, Jinghui Wang, Guoan Zhu

**Affiliations:** Faculty of Materials and Energy, Southwest University, Chongqing 400715, China; xzjlms13@email.swu.edu.cn (Z.X.); chen_zq@swu.edu.cn (Z.C.); cheng_np@swu.edu.cn (N.C.); w1197757910@email.swu.edu.cn (J.W.); zhu970504@email.swu.edu.cn (G.Z.)

**Keywords:** nanotube, tunable bandgap, optical property, electric field, black phosphorus

## Abstract

Black phosphorus (BP), a new two-dimensional material, has been the focus of scientists’ attention. BP nanotubes have potential in the field of optoelectronics due to their low-dimensional effects. In this work, the bending strain energy, electronic structure, and optical properties of BP nanotubes were investigated by using the first-principles method based on density functional theory. The results show that these properties are closely related to the rolling direction and radius of the BP nanotube. All the calculated BP nanotube properties show direct bandgaps, and the BP nanotubes with the same rolling direction express a monotone increasing trend in the value of bandgap with a decrease in radius, which is a stacking effect of the compression strain on the inner atoms and the tension strain on the outer atoms. The bending strain energy of the zigzag phosphorene nanotubes (zPNTs) is higher than that of armchair phosphorene nanotubes (aPNT) with the same radius of curvature due to the anisotropy of the BP’s structure. The imaginary part of the dielectric function, the absorption range, reflectivity, and the imaginary part of the refractive index of aPNTs have a wider range than those of zPNTs, with higher values overall. As a result, tunable BP nanotubes are suitable for optoelectronic devices, such as lasers and diodes, which function in the infrared and ultra-violet regions, and for solar cells and photocatalysis.

## 1. Introduction

Two-dimensional (2D) nanomaterials are one of the most attractive research topics due to their outstanding potential applications in many fields, such as flexible electronics [1], sensing [2], and optics [3], as a result of their desirable physical and structural properties [4,5,6,7]. Among the 2D materials, graphene is outstanding as it has the highest charge carrier mobility [8], but fails to act as a semiconductor due to a lack of bandgap in its electronic structure [9]. This deficiency hinders its use in many applications, especially for optoelectronic devices. Transition metal dichalcogenides (TMDCs), another main member in the 2D material family, have also attracted the interest of scientists. Molybdenum disulfide (MoS_2_), the most common TMDC [10], has a noticeable bandgap, enabling the conversion of electrons into photons of light and resulting in extraordinary on/off ratios (>10^8^) [11]. However, the sandwich structure of MoS_2_ restricts its charge carrier mobility [12]. Phosphorene, a monolayer or few-layer material, appears to be adesirable alternative.

Black phosphorus (BP), one of the three main allotropes of phosphorus, is thermodynamically the most stable compared to its red and white counterparts. One of the extraordinary properties of black phosphorus is its high mobility [13,14,15,16,17], which is responsible for a relatively large part of BP’s unique electronic properties. Previous studies showed few-layer BP possesses high mobility, ranging from 600 cm^2^/Vs to 1000 cm^2^/Vs at room temperature [14,15,16,18], allowing BP to be applied in electrode materials. Additionally, another outstanding property of BP is its direct and tunable bandgap in both mono- and multi-layered forms [14,15] making BP an ideal semiconductor for potential applications in extraordinary light emission and efficient photo-electrical conversion. Furthermore, due to the small bandgap attributed to the large excitation binding energy, both p-type and n-type configurations can be tuned in BP to satisfy the wide range of demands in optoelectronic devices, including the tubular devices.

BP thin films have been receiving the attention of scientists worldwide. The puckered structure provides BP with in-plane anisotropy, resulting in its thermal conductivity [19] and unique angle-dependent transport anisotropy [20], especially in carrier mobility [21,22]. Based on the special properties of black phosphorus, the characteristics of BP nanotubes have attracted more attention [23,24,25], as have carbon nanotubes. Comparatively, the research on BP tubes is limited. However, the urgent demand for tubular electronics stresses the importance of BP tube research, especially for its electronic and optical properties. Because of the anisotropy of black phosphorus, the rolling direction of BP nanotubes is a determining factor of their electrical properties. Additionally, the bandgap and optical properties of BP nanotubes can be modulated by their diameter. Therefore, we systematically studied the properties of BP tubes with two different rolling directions and various radii.

## 2. Computational Models and Method

### 2.1. Model Construction

**Figure 1:**
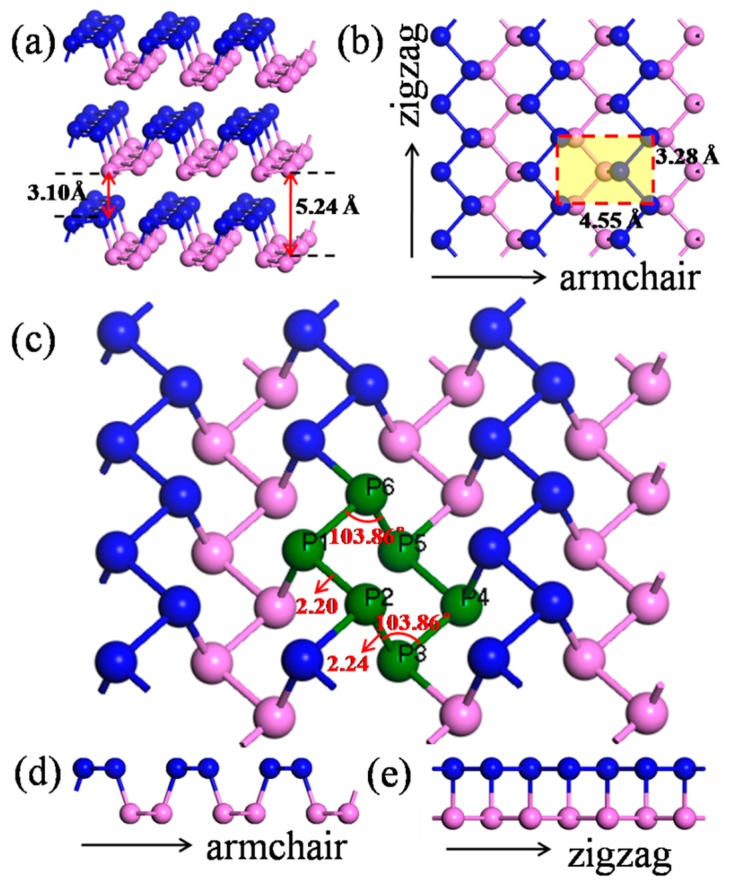
The structures of black phosphorus (BP): (**a**) bulk BP; (**b**) top view of monolayer BP; (**c**) computing unit of the puckered orthorhombic lattice; (**d**) side view from the armchair direction; and (**e**) side view from zigzag direction. Atoms in the top and bottom layers are in blue and pink, respectively.

Similar to graphene, bulk black phosphorus is a layered material composed of individual stacking atomic layers by van der Waals interactions. Figure 1 illustrates the structure of black phosphorus from different side views. Since there are five electrons on the 3p orbitals of the phosphorus (P) atom, within the phosphorene structure, each P atom covalently bonds with three adjacent P atoms with a 2.20 Å or 2.24 Å bond length in the monolayer model, leaving one lone pair [20], forming a puckered orthorhombic lattice [23,24], resulting in a layer-to-layer spacing of approximately 5 Å [20,25]. The corresponding parameters are shown in Table 1. Given the asymmetrical crystal structures caused by the corrugated pattern of phosphorus atoms, BP has different properties compared to graphene and TMDCs. In calculations, a separation of 30 Å, 30 Å, 40 Å, 40 Å, and 50 Å for the adjacent nanotubes with radii of 5.2 Å, 7.3 Å, 10.06 Å, 11.5 Å, and 12.85 Å, respectively, was used to minimize the mirroring interaction.

**Figure 2:**
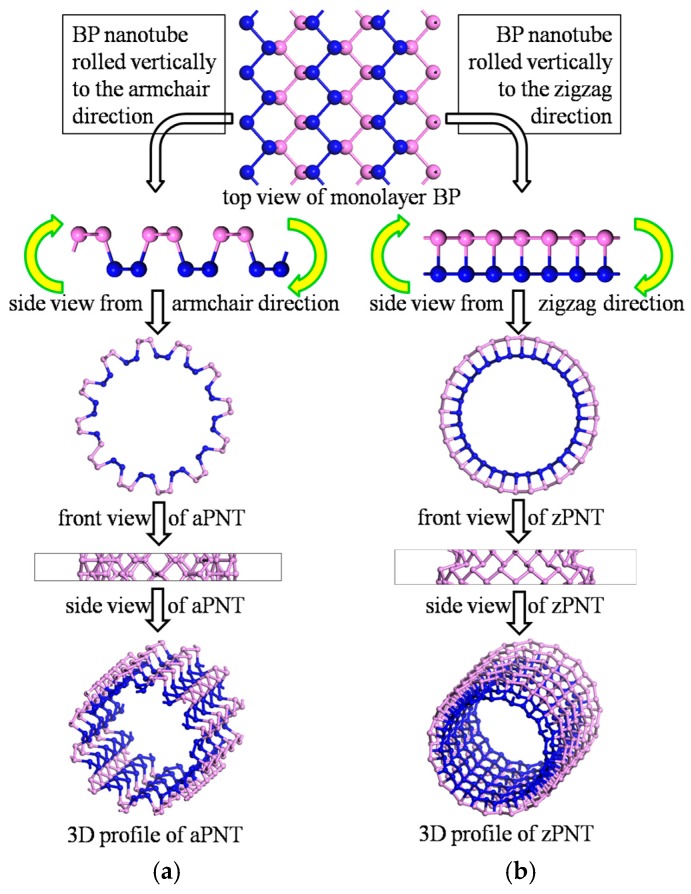
Schematic diagram of the construction of BP nanotubes.

Figure 2 presents a flow diagram for constructing BP nanotubes. Based on the puckered structure of BP, BP nanotubes are separately rolled vertically to the zigzag or armchair direction to construct the nanotubes correspondingly named armchair phosphorene nanotubes (aPNT) (Figure 2a) and zigzag phosphorene nanotubes (zPNT) (Figure 2b). In addition to the rolling direction factor, the radius of curvature was also considered. As a result, the BP nanotubes were built with different radii of 5.2 Å, 7.3 Å, 10.06 Å, 11.5 Å, and 12.85 Å, to analyze the effect of the bending strain energy on the electronic and optical properties. The radius values are consistent with the middle circles of the models, and non-integral values were chosen to ensure the integrity of the atomic periodicity of the puckered structure.

### 2.2. Energy Calculation Method

All the calculations were performed using the pseudo-potential plane-wave method [29] and implemented in the Cambridge Serial Total Energy Package Program (CASTEP; Materials Studio 8.0, Accelrys, San Diego, CA, USA) [30]. The electronic exchange-correlation energy was determined using the generalized gradient approximation of Perdew-Burke-Ernzerh (GGA-PBE) [29]. All nanotubes were fully relaxed with respect to the volume and all cell-internal atomic coordinates. The convergence of the results with respect to energy cutoff and *k*-points was carefully considered. A plane-wave basis set was used with an energy cutoff of 600 eV. We used 1 × 1 × 32 and 1 × 1 × 24 Monkhorst-pack *k*-point meshes for aPNT and zPNT with a spacing of 0.01 nm^−1^ for all calculations. All atomic positions were optimized using the Broyden-Flecher-Goldfarb-Shanno (BFGS) scheme based on the cell optimization criterion. The convergence was confirmed with the system total energy fluctuation within 5 × 10^–6^ eV. The force on each atom in the unit cell was less than 0.01 eV/Å, the residual stress of the unit cell was lower than 0.02 GPa, and the tolerance offset was lower than 5 × 10^−6^ Å.

## 3. Results and Discussion

### 3.1. Bending Strain Energy

Bending strain energy is defined as the energy difference per atom between the nanotube models and the planar monolayer BP, which can be calculated as follows:

(1) $$E_{b}=\frac{E_{\mathrm{nt}}-NE_{s}}{N}$$

where *E*_b_ is the bending strain energy, *E*_nt_ is the total energy of nanotubes, *E*_s_ is the energy of the planar monolayer BP per atom, and *N* is the number of BP atoms in the calculated unit cell.

The formation energy per atom of the PNTs can be obtained from the following equation:

(2) $$E_{f}=\frac{NE_{a}-E_{\mathrm{nt}}}{N}$$

where *E*_f_ is the formation energy, and *E*_a_ is the energy per free P atom.

**Figure 3:**
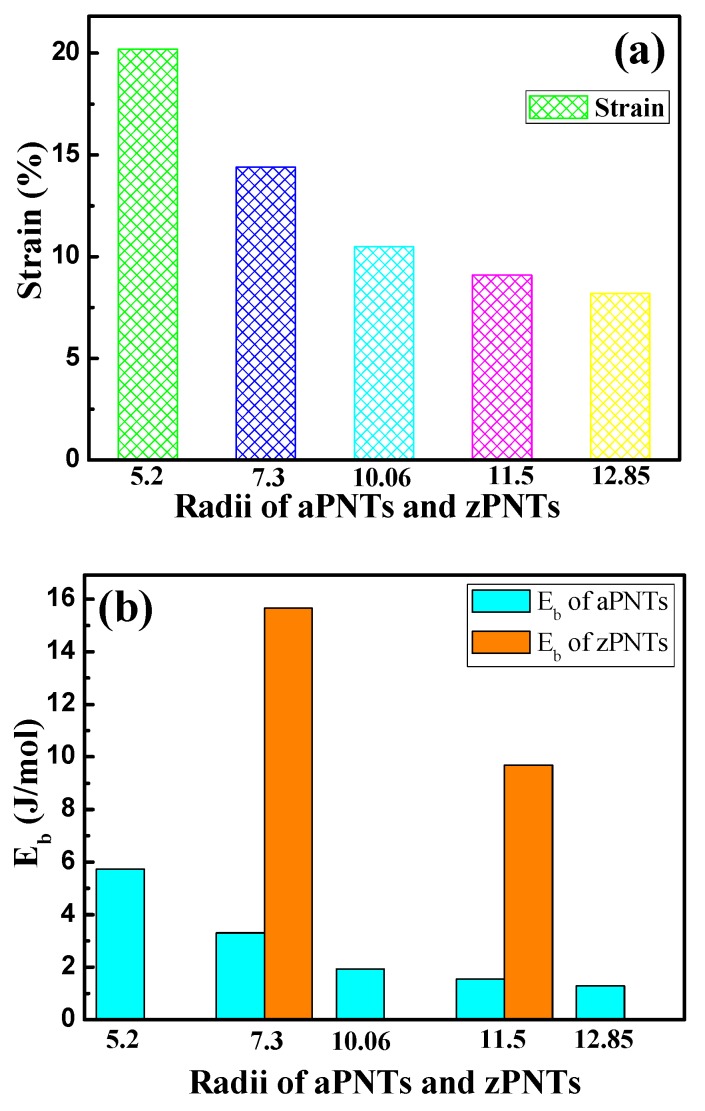
(**a**) Bending strain and (**b**) bending strain energy of aPNTs with different bending directions and radii.

The calculated results of the bending strain energy and formation energy are shown in Table 2 and Figure 3. As shown in Figure 3a, the bending strain energy of the BP nanotubes monotonically increases with the decrease in the models’ radii with the same bending direction. The reason for this variation is that when the BP planar monolayer is rolled into a nanotube, the puckered structure of BP forms a nanotube with a specified thickness, including inner atoms located in the inner wall and outer atoms on the ektexine of the nanotube. The different radii of the inner wall and ektexine result in different stress modes, with compressive strain on the inner atoms and tension strain on the outer atoms. The compressive and tensile strain on the inner and outer atoms can be expressed as the ratio of thickness to diameter, as shown in Figure 1 and Equation (3).

(3) $$S=\frac{h/2}{r}=h/d$$

where *h* is the thickness of the planar monolayer BP, and *r* and *d* represent the radius and diameter of the nanotube, respectively. With the decrease in the nanotube radius, the rate of the compressive strain on the inner atoms and tension strain on the outer atoms both increase, leading to amonotone increase in the bending energy shown in Figure 3a.

Furthermore, the bending strain energy of the zPNTs is higher than that of the aPNTs with the same radii of curvature, due to the anisotropy of the BP structure. As shown in Figure 1 and Table 3, in one structural unit, three bonds have a bond length of 2.242 Å and four bonds have a bond length of 2.203 Å. The lattice constant along the armchair is much larger than that along the zigzag direction in the BP periodic unit shown in Figure 1. Along the armchair direction, bonds with a bond length of 2.242 Å dominate the strain of the structure and release the compressive or tension strain by changing the angle between the adjacent bonds which partially releases the compressive and tensile stress between the inner and outer atoms and results in a smaller distortion of the bond length between the adjacent atoms, as shown in Table 3. However, for zPNTs, the bending strain during rolling is mainly imposed on the shorter bonds with a bond length of 2.203 Å as shown in Figure 1. Generally, a shorter covalent bond length means a greater cohesive strength, which can be theoretically deduced from the bond population shown in Table 3. So, for zPNTs, despite experiencing the same strain, both the compressive strain energy under gone by the inner wall atoms and the tension strain energy experienced by the ektexine atoms are higher than those of the atoms in aPNTs. Therefore, much larger deviations in bond lengths are imposed on the zPNTs atoms than those in aPNTs, as shown in Table 3. As a result, the bending strain energy of the zPNTs is much higher than that of the aPNTs with the same curvature radius. The higher the bending strain energy, the greater the instability. This is the reason why the zPNTs with a radius under 7.3 Å cannot achieve energy convergence during the optimizing process. The formation energy indicates the strength of atomic bonding and reflects the stability of the phase. The higher the value of the formation energy, the more stable the structure. In Table 2, the formation energy of zPNT with radius of 11.5 Å is even lower than that of aPNT with radius of5.2 Å. As a result, the aPNTs are more stable than the zPNTs with the same radius.

### 3.2. Electronic Properties

#### 3.2.1. Bands Structure

**Figure 4:**
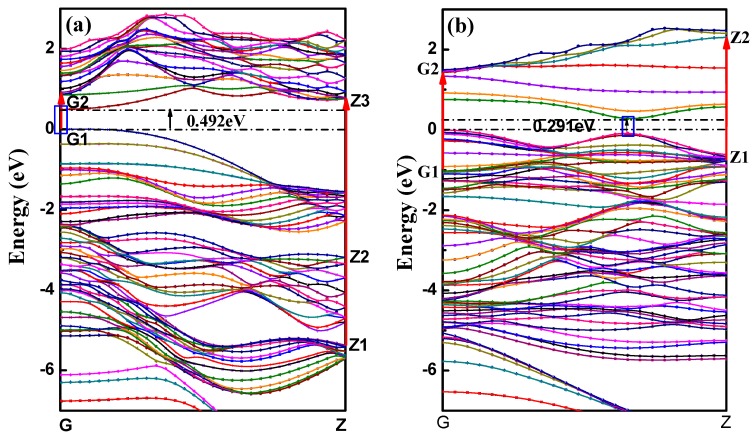
Band structures of BP nanotubes with a radius of 7.3 Å: (**a**) aPNT and (**b**) zPNT. The red arrows represent the inter-band transitions between the valence and conduction bands, and the black arrows point out the band-gaps of band structures of aPNT and zPNT.

All the calculated BP nanotubes showed direct bandgaps, with the top of valence band located at the same site with the bottom of the conduction band in reciprocal space, which is consistent with the planar monolayer BP characteristics. This means the BP nanotube can directly couple with light, making BP an ideal semiconductor for potential applications in extraordinary light emission and efficient photo-electrical conversion. The band structure of aPNT and zPNT with a radius of 7.3 Å are shown in Figure 4.

**Figure 5:**
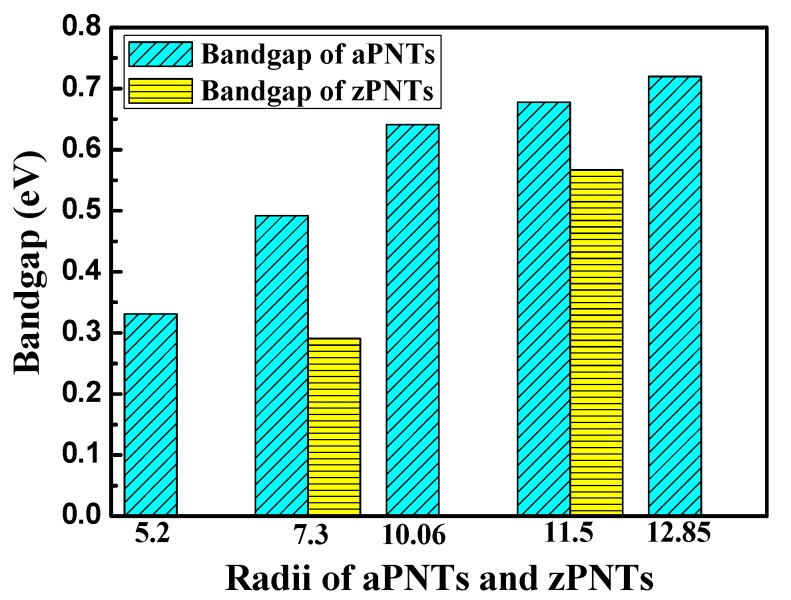
The variation in bandgaps for aPNTs and zPNTs with different radii.

**Figure 6:**
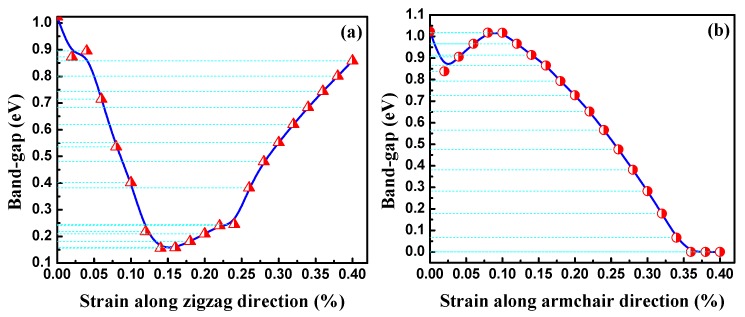
Band-gaps of monolayer phosphorus under different uniaxial compressive and tensile stresses along (**a**) zigzag and (**b**) armchair directions.

The bandgaps of the BP nanotubes with different rolling direction and radii are shown in Figure 5. BP nanotubes in the same rolling direction demonstrate a monotone increasing trend in the bandgaps with a decrease in radius, which is consistent with a previous finding [24]. When the radius of the nanotube is sufficiently large, its bandgap value should approach to that of the planar BP monolayer. The trend is consistent with that of the bending strain energy. With higher bending strain energy, the BP nanotubes in the same rolling direction exhibit a narrower bandgap. This is the stacking effect of the compression strain on the inner atoms and the tension strain on the outer atoms. To further explain the influence of stress on the BP bandgap, uniaxial tension and compression were placed on the planar monolayer BP. Figure 6 shows the effects of the uniaxial compressive and tensile stress along the armchair and zigzag directions on the bandgaps. With compressive strain or tension strain along zigzag direction, the bandgaps of the modes all monotonically decreased with a large slope. Therefore, when the BP monolayers were rolled into nanotubes along the armchair direction, the compressive strain of the inner atoms and the tension strain of the outer atoms induced a decrease in their bandgaps. A smaller radius results in a larger bending strain energy, leading to a narrower bandgap.

When the nanotubes were rolled in the zigzag direction, the strain energy imposed on the atoms was more severe, as discussed in the previous section. With the same radius, the zPNTs have narrower bandgaps than the aPNTs. Under tensile strain, the variation in the bandgap trend of the planar monolayer BP was different, quickly declining followed by a gradual recovery. With tension at 14%, the bandgap reached a valley with a value of about 0.15 eV. With compressive strain, the bandgap of the zPNT decreased slowly compared to the aPNT, as shown in Figure 5. Because of this, the rate of decrease of the bandgap did not keep pace with the increase in the bending strain energy shown in Table 2, though the bandgap of the zPNT was narrower than that of aPNT with the same radius.

#### 3.2.2. Electron Density and Difference in Electron Density 

**Figure 7:**
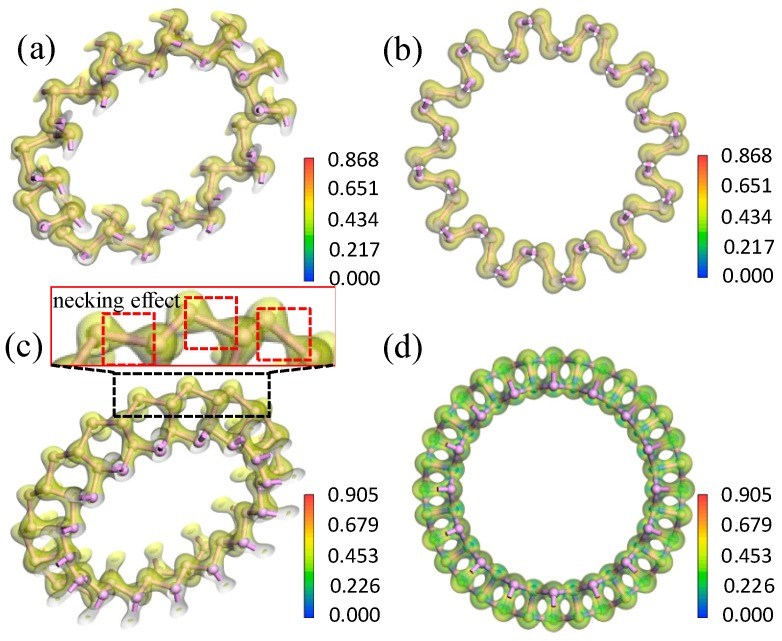
Electron density diagrams for (**a**,**b**) aPNT and (**c**,**d**) zPNT with a radius of 7.3 Å. (**a**) The rotated aPNT with electron density isosurface to show the weak difference in the electron distribution around the P atoms located at the inner and outer rings of Apnt; (**b**) The electron density profile vertical to the central axe of aPNT; (**c**) the rotated zPNT with electron density isosurface to show the necking effect shown around the P atoms located at the outer ring of zPNT and (**d**) the electron density profile vertical to central axe of zPNT.

**Figure 8:**
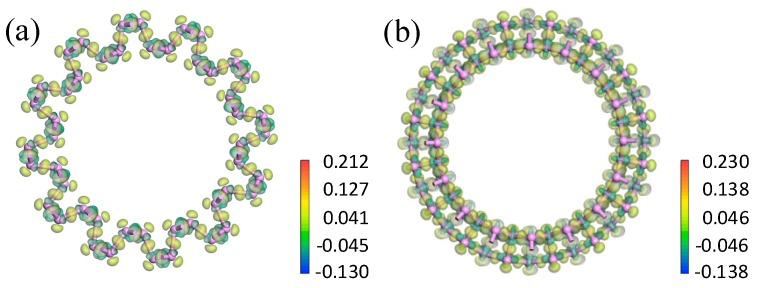
Electron density difference for aPNT and zPNT with a radius of 7.3 Å. The profiles of the electron density difference vertical to the central axe of (**a**) aPNT; and (**b**) zPNT. The attached rulers show the transferred charge corresponding to the colors.

To further analyze the influence of bending direction and curvature radius on the bending strain energy and bandgap, the electron density and electron density difference were investigated. From the electron density shown in Figure 7, the isosurfaces of the electron density demonstrated strong covalent bonding between the P atoms. Only a small quantity of electrons transferred between neighboring P atoms. As shown in Figure 8, the maximum transferred charge for aPNT with a radius of 7.3 Å was only 0.212, and for zPNT with radius of 7.3 Å was only 0.230. Furthermore, all electrons shifted from the periphery of the atom to the bond direction, demonstrating the strong covalent interaction between the P atoms once again. As shown in Table 3, the zPNTs distorted more than the aPNTs with the same radius. So, with the increase in the distance between the two neighboring atoms located at the ektexine in zPNTs (Figure 7), the necking phenomenon was observed on the isosurface of electron density, meaning a weakened interaction occurs under tensile strain. Furthermore, we deduced that the aPNTs would be more stable than the zPNTs because both the inner atoms with a bond length 2.19 Å and the outer atoms with a bond length of 2.23 Å have much higher bond populations, at 1.05 and 0.87, respectively, than the corresponding values of 0.36 and 0.32, in zPNTs, shown in Table 3. This reflects the higher stability of aPNTs than zPNTs based on the bond strength.

### 3.3. Optical Properties

Notably, due to the in-plane anisotropic structure of the BP thin film, the angle-resolved polarized, or the polarization-resolved, optical property is considered an effective technique to investigate the crystalline orientation of the sample [26,27,28]. The dependence of the anisotropic optical spectrum on incident waves helps to determine crystallographic orientation. As a result, sufficient caution is required when the optical spectrum is used for BP nanotubes with different rolling directions and radii. Alternatively, the anisotropic optical absorption spectroscopy along the armchair and zigzag directions of the BP crystals should result in diverse optical properties of aPNTs and zPNTs.

#### 3.3.1. Complex Dielectric Constants

The optical properties of solids can be obtained through electronic transitions. Within the range of the linear optical response, the complex dielectric function, $\varepsilon (\omega )=\varepsilon_{1}(\omega )+i\varepsilon_{2}(\omega )$ always represents the macroscopic optical response function in a solid, where $\varepsilon_{1}(\omega )$ is the real component of the dielectric function, and $\varepsilon_{2}\left(\omega \right)$ isits imaginary part. The complex dielectric function can be calculated from the momentum matrix elements of occupied and unoccupied wave functions according to Equation (4) and can be thought of as detailing the real transition between occupied and unoccupied electronic states.

(4) $$\varepsilon_{2}(\omega )=\frac{4\pi^{2}}{m^{2}\omega^{2}}\cdot \sum_{V,C}\int_{BZ}d^{3}k\frac{2}{2\pi }|e\cdot M_{CV}{\left(K\right)|}^{2}\times \left[E_{C}(K)-E_{V}(K)-\hbar \omega \right]$$

where *e* is the electric quantity of an electron, *ω* is the frequency of the incident photon, *C* and *V* are the conduction and valence bands, respectively, *E_C_*(*K*) and *E_V_*(*K*) represent the intrinsic energy of the conduction band and the valence band, respectively, *BZ* is the first Brillouin zone, *K* is the reciprocal lattice vector, and $|e\cdot M_{CV}\left(K\right)|$ is the momentum matrix element [18]. According to the Kramer-Krönig dispersion relationship, the real part of the dielectric function can be obtained by Equation (5):

(5) $$\varepsilon_{1}(\omega )=1+\frac{8\pi e^{2}}{m^{2}}\cdot \sum_{V,C}\int_{BZ}d^{3}K\frac{2}{2\pi }\frac{|e\cdot M_{CV}{\left(K\right)|}^{2}}{\left[E_{C}(K)-E_{V}(K)\right]}\times \frac{\hbar^{3}}{{\left[E_{C}(K)-E_{V}(K)\right]}^{2}-\hbar^{2}\omega^{2}}$$

**Figure 9:**
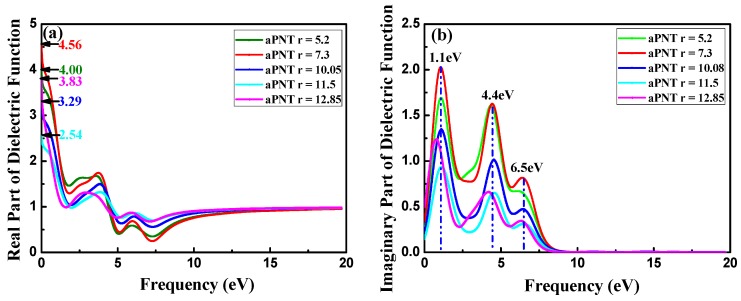

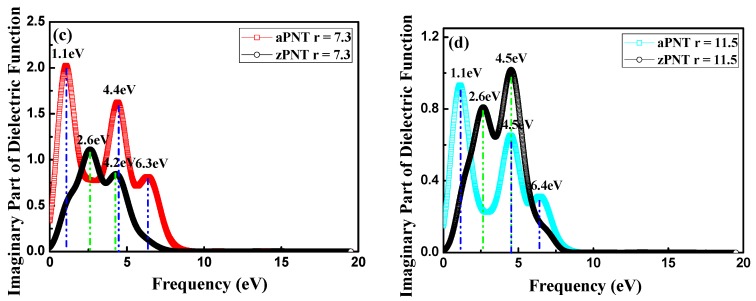
Dielectric functions: (**a**) Real (Re) part and (**b**) imaginary (Im) part for aPNTs with different radii; (**c**) Real (Re) and (**d**) imaginary (Im) parts of the dielectric function for aPNTs and zPNTs with the same radius.

The real and imaginary parts of the dielectric function, which are tightly connected with band structure, for aPNTs and zPNTs with different radii are shown in Figure 9. The differences in the dielectric function of aPNTs and zPNTs originate from the anisotropy of the 2D nature of the atomic configuration. The static dielectric constant (the value of dielectric constant at zero frequency *ε*(0)) changes with the rolling direction and the radius of the BP nanotubes. Along with the decrease in the aPNTs radius, the static dielectric constant fluctuates, but the aPNTs static dielectric constant is higher than that of zPNTs. The dielectric constant under high frequency *ε*(∞) tends to become a constant at 0.8. Three peaks exist in the imaginary part of aPNTs; however, only two peaks appear in that of zPNTs. The imaginary part of the dielectric function, in the energy range of 0 to 20 eV for BP nanotubes, are clearly related to their band structure, indicating the absorption behavior, so that the electronic transitions from valance to conduction bands contribute to the main part of the optical spectra.

The threshold energies of the dielectric function correspond to the system bandgaps. The threshold energy for the transition between the highest valence band and the lowest conduction band is the fundamental absorption edge. The peaks are related to different electronic transitions from occupied states (valence bands) to the unoccupied states (conduction bands). The highest peak in the imaginary part of aPNTs at a radius of 7.3 Å is located at 1.1 eV, which shows a slight red shift with an increase in radius. The other two peaks are situated at 4.4 eV and 6.5 eV. These three peaks are related to the inter-band transitions between the valence and conduction bands, from G1 to G2 at the high-symmetry point G in the reciprocal space, from Z1 to Z3 at the high-symmetry point Z, and from Z2 to Z3 at the high-symmetry point Z (Figure 4a). Comparatively, the two peaks in the imaginary part of zPNTs are located at 2.6 eV and 4.2 eV, attributed to the inter-band transitions from G1 to G2 at the high-symmetry point G, and Z1 to Z2 at the high-symmetry point Z (Figure 4b). The lower energy values are caused by the near-band electronic direct transitions. Comparing the imaginary parts of aPNTs with different radii, the peak values gradually decline with an increase in the radius. The peak shape broadens as a result of the changes in the band structure and the density of the different states.

#### 3.3.2. Absorption Coefficient

The optical absorption spectrum is important for estimating the optical properties of a material to be used as an optoelectronic device. The absorption coefficient indicates the fraction of energy lost by the wave when it passes through a material. The absorption coefficient I(ω) can be derived from the real part $\varepsilon_{1}(\omega )$ and imaginary part $\varepsilon_{2}(\omega )$ of the dielectric function as follows:

(6) I(ω)=2ω(ε1(ω)2−ε2(ω)2−ε1(ω))12

**Figure 10:**
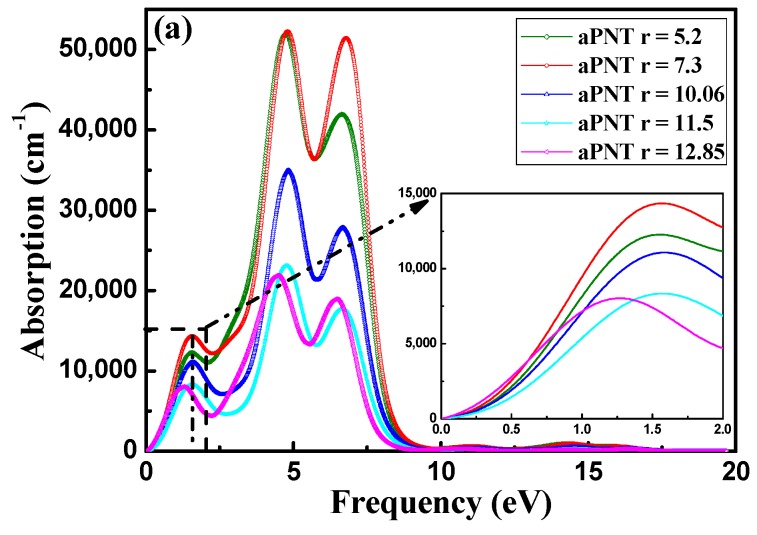

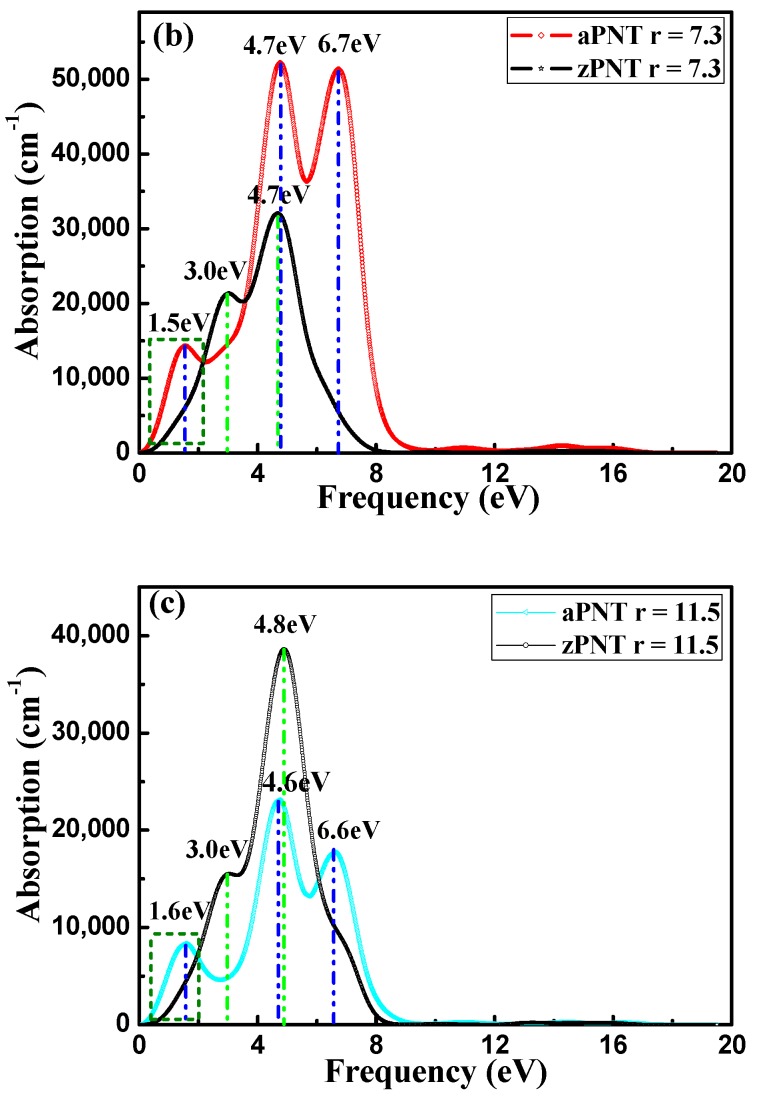
Absorption coefficient of BP nanotubes with electric field direction parallel to the radius. (**a**) aPNTs with different radii; (**b**) aPNT and zPNT with a radius of 7.3 Å, and (**c**) aPNT and zPNT with a radius of 11.5 Å.

For BP nanotubes, the absorption coefficient is related to both the extinction index and the imaginary part of the dielectric function (Figure 10). The absorption spectra of aPNT and zPNT at a radius of 7.3 Å in Figure 10b are obviously different. The absorption range of aPNT is much larger than that of zPNT, and aPNT has a higher absorption coefficient. As a result, the aPNTs are more efficient for the absorption of low frequency incident light. For aPNTs, the first absorption peak occurs at an energy of 1.5 eV, and the highest absorption peak is located at an energy of 4.7 eV. However, for zPNTs, only two peaks appear in the absorption spectrum, with the first peak occurring at 3.4 eV and the highest peak appearing at 4.7 eV. The visible light region ranges from 1.62 eV to 3.11 eV. According to Figure 10b, aPNTs have a better absorption from the infrared region to the visible region, and even to the ultra-violet region, which is reconfirmed by comparing the absorption index of aPNT and zPNT at a radius of 11.5 Å as shown in Figure 10c. So, BP nanotubes are suitable for optoelectronic devices such as lasers and diodes that work in the infrared and ultra-violet regions, and are also appropriate for solar cells and photocatalysis.

**Figure 11:**
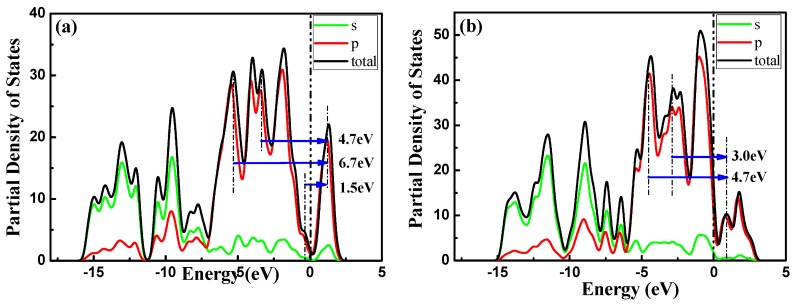
Partial Density of States (PDOS) of BP nanotubes with radius of 7.3 Å: (**a**) aPNTs and (**b**) zPNTs.

These peaks could originate from the transition of covalence bands to conduction bands, as discussed in the imaginary part of the dielectric function in Section 3.3.1. This can also be explained by the partial density of states (PDOS). As shown in Figure 11, the transition of 2s-P (occupied) and 2p-P (occupied) states into 3p-P (unoccupied) states in the PDOS of aPNTs results in the absorption peak at 1.5 eV. Similarly, the peaks at 4.7 eV and 6.7 eV originate from the transition of 2s-P (occupied) and 2p-P (occupied) states into 3p-P (unoccupied) states, as shown in Figure 11.

The absorption edge $E_{op}$ can be calculated using Equation (7):

(7) $$\alpha hv=C{\left(hv-E_{op}\right)}^{n}$$

where *hυ* is the photo energy, *C* is a constant, and *α* is the absorption coefficient. For materials with a direct bandgap, such as the BP nanotubes, *n* is 0.5; for materials with an indirect bandgap, *n* is set to 2.

The absorption edge can be determined using the linear extrapolation method, and the results were 0.5 eV for aPNT and 0.3 eV for zPNT with a radius of 7.3 Å, which corresponds to the values of the bandgap between the highest valance band and the lowest conduction band, at 0.492 eV and 0.291 eV, as shown in Table 2. Additionally, the absorption edge shows a slight red shift with a decrease in the radius of the nanotubes, which is also consistent with trend in the bandgap value.

Comparing the absorption spectra of aPNTs with different radii (Figure 10), all the absorption peaks of aPNTs monotonously decline with the increase in the radii of the BP nanotubes as long as the radius is bigger than 7.3 Å.

#### 3.3.3. Reflection Coefficient

The reflectivity at normal incidence is given by:

(8) $$R(\omega )=\frac{{\left(n-1\right)}^{2}-k^{2}}{{\left(n+1\right)}^{2}+k^{2}}={\left|\frac{\sqrt{\varepsilon_{1}(\omega )+i\varepsilon_{2}(\omega )}-1}{\sqrt{\varepsilon_{1}(\omega )+i\varepsilon_{2}(\omega )}+1}\right|}^{2}$$

where *n* and *k* are the real and imaginary parts of the complex refractive index, which are known as the refractive index and the extinction index, respectively, and are given by the following relationship:

(9) $$n(\omega )=\sqrt{\frac{\left|\varepsilon (\omega )+\varepsilon_{1}(\omega )\right|}{2}}=\sqrt{\frac{\sqrt{\varepsilon_{1}^{2}(\omega )+\varepsilon_{2}^{2}(\omega )}+\varepsilon_{1}(\omega )}{2}}\;k(\omega )=\sqrt{\frac{\left|\varepsilon (\omega )-\varepsilon_{1}(\omega )\right|}{2}}=\sqrt{\frac{\sqrt{\varepsilon_{1}^{2}(\omega )+\varepsilon_{2}^{2}(\omega )}-\varepsilon_{1}(\omega )}{2}}$$

**Figure 12:**
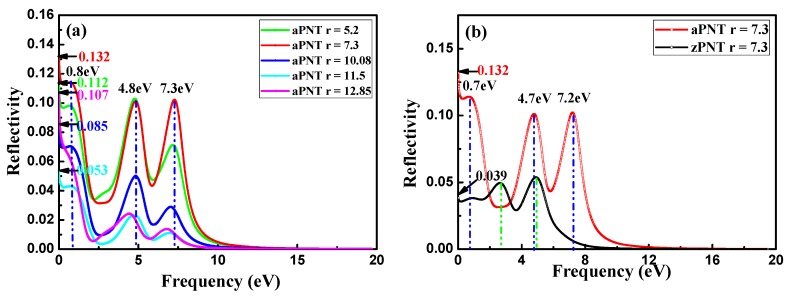

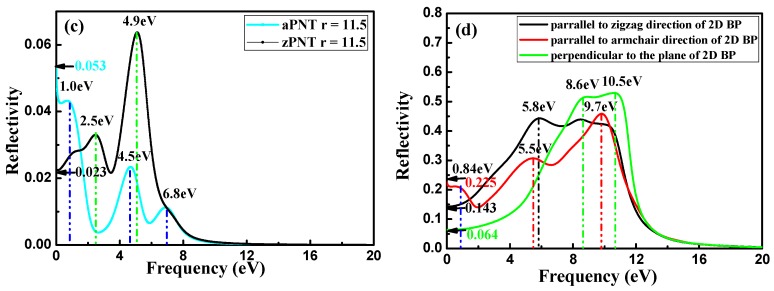
Reflectivity of nanotubes and two-dimensional (2D) BP: (**a**) aPNTs with different radii; (**b**) aPNT and zPNT with the same radius of 7.3 Å; (**c**) aPNT and zPNT with the same radius of 11.5 Å, and (**d**) reflectivity of 2D BP along different polarizations.

The reflectivities of aPNTs and zPNTs with perpendicular polarization are shown in Figure 12. Reflectivity is sensitive to the rolling direction and the radius of PNTs, including the static reflectivity. Comparing Figure 12 to Figure 10, the reflectivity peaks are located at the same sites as those of the absorption coefficients if the frequency of the incident light is higher than the value of the first peak of the absorption coefficient. This is because a solid can effectively reflect the light in the same scope if it shows a strong absorption fora specific light range. For the aPNTs shown in Figure 12a, when the frequency is lower than the value of the first peak of the absorption coefficient, the reflectivity sharply increases, resulting in the intense reflection of low frequency incident light because of the smaller transition from the valence band to the conduction band.

Conversely, zPNTs do not exhibit any sign of increase at the beginning of the increase in frequency, as shown in Figure 12b,c. For aPNTs, after a drop in the static reflectivity (0–0.1 eV) and at the beginning of the reflectivity curves, a platform gently increases (ranging from 0.1 to 0.8 eV) followed by a decrease from 0.8 to 2.5 eV. Instead, for zPNTs, an evident increase occurs from 0 to 1 eV, and then the value is maintained within a narrow range. In the 2D black phosphorene layer, the optical reflectivity spectra display a strong directional dependency (Figure 12d). These characteristics correspond to the different variations in the reflectivity of 2D black phosphorus with its polarization direction along armchair or zigzag directions. The peaks in the aPNTs reflectivity curves show an overall red shift, due to the strain sustained by the nanotubes. Additionally, aPNTs exhibit a significantly higher reflectivity to low frequency incident light. Overall, the reflectivity of aPNTs is higher than that of zPNTs, except at low frequencies in the range of 2.0 to 3.2 eV for PNTs with radius of 7.3 Å, and from 0 to 1.75 eV for PNTs with radius of 11.5 Å (Figure 12b,c). For aPNTs, three peaks around 0.8, 4.8, and 7.3 eV in the reflectivity curves show a red shift relative to the 2D reflectivity polarized along the armchair direction. This is due to the narrowed bandgap shown in Figure 5, which is induced by the stains the nanotubes sustained during their formation process. Correspondingly, a similar conclusion can be drawn for zPNT.

#### 3.3.4. Refractive Index

The refractive index can be expressed as:

(10) $$N(\omega )=n\left(\omega \right)+ik\left(\omega \right)\;n{\left(\omega \right)}^{2}-k{\left(\omega \right)}^{2}=\varepsilon_{1}\left(\omega \right)+i\varepsilon_{2}\left(\omega \right)\;2n\left(\omega \right)k\left(\omega \right)=\varepsilon_{2}\left(\omega \right)$$

**Figure 13:**
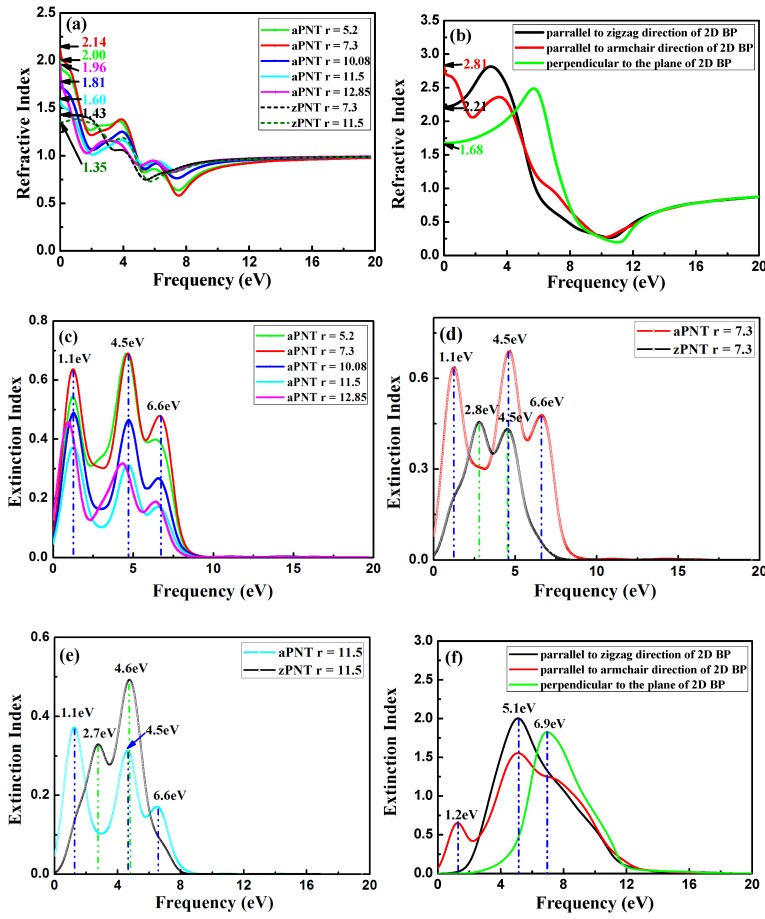
Refractive index of nanotubes with different radii and 2D BP: (**a**) refractive index *n* of aPNTs with different radii; (**b**) refractive index *n* of 2D BP along different polarizations; (**c**) extinctive index *k* of aPNTs with different radii; (**d**) extinctive index *k* of aPNT and zPNT with the same radius of 7.3 Å; (**e**) extinctive index *k* of aPNT and zPNT with the same radius of 11.5 Å, and (**f**) extinctive index *k* of 2D BP along different polarizations.

The refraction index *n* and extinction index *k* for BP nanotubes, calculated according to Equation (9), are shown in Figure 13. The static refractive index shows its dependence on the rolling direction and the radius of PNTs, which fluctuates with the change in the radius of nanotubes, similar to the dependence on the change in static dielectric function shown in Figure 9a. In addition, the static refractive indexes of aPNTs, ranging from 1.6 to 2.2 eV, are higher than those of zPNTs, ranging from about 1.3 to 1.5 eV as a whole, attributed to the larger static refractive index of 2D BP with polarization parallel to the armchair direction shown in Figure 13b, compared to the values for polarization parallel to the zigzag direction. The refractive indexes of aPNTs and zPNTs in the low frequency range show completely different trends. Similar to the reflectivity, with increasing frequency, the refractive index *n* of aPNTs sharply decrease when the frequency starts to increase, and then maintains a gentle decrease, followed by a sharp decrease (Figure 13a). This corresponds to the variation in the refractive index for 2D BP polarized along the armchair direction as shown in Figure 13b. The reverse change in zPNTs compared to aPNTs for low frequencies is also attributed to the variation in the refractive index for 2D BP polarized along the zigzag direction. Additionally, for aPNTs, the real parts of the refraction index *n* have the lowest values at 1.1, 4.5, and 6.6 eV, which correspond to the peaks of the imaginary parts of refractive index *k*. This is consistent with the peaks of the absorption coefficient shown in Figure 10, because the extinction index *k* represents the decrease in the incident light as it propagates through the material. The extinctive index is different because three peaks are distributed around 1.1, 4.5, and 6.6 eV for aPNTs; however, only two peaks appeared in the extinction curves of zPNTs. This is because the extinction indexes of aPNTs have a combined effect for the extinction peaks appearing in the extinction index of 2D BP with polarization along the armchair direction (2.2 and 6.1 eV) and with polarization perpendicular to the 2D BP plane (7.9 eV). Similar to the reflectivity of aPNTs, the red shift of the peaks in the extinction indexes for aPNTs and zPNTs are a result of the narrowed bandgaps under strain.

## 4. Conclusions

Based on density functional theory, the bending strain energy, electronic structure, and optical properties of BP nanotubes were investigated. The results showed that these properties are closely related to the rolling direction and radius of the BP nanotube. The bending strain energy of the zPNTs is considerably higher than that of the aPNT with the same curvature radius, due to the anisotropy of the BP's structure, showing that the aPNTs are more stable than the zPNTs with the same radius. Additionally, the bending strain energy of BP nanotubes monotonically increases with the decrease in the models' radius with the same bending direction. All the calculated BP nanotubes showed direct bandgaps, and the BP nanotubes with the same rolling direction expressed a monotone increasing trend in the value of the bandgap with the decrease in radius, which is a stacking effect of the compression strain on the inner atoms and the tension strain on the outer atoms. Additionally, for the zPNTs, the nanotubes rolled along the zigzag direction, and experienced more severe strain on the lattice than aPNTs. The zPNTs showed narrower bandgaps than the aPNTs with the same radius.

The imaginary part of the dielectric function, the absorption range, reflectivity, and the imaginary part of the refractive index of aPNTs had a wider range than those of zPNTs, having higher values overall. The absorption edge of aPNTs had a higher value than that of zPNTs with the same radius, which corresponds to the bandgap values. As a result, the aPNTs are more efficient for the absorption of low frequency incident light. Additionally, all of the absorption peaks of aPNTs monotonously decreased with the increase in the radius of BP nanotubes as long as the radius was larger than 7.3 Å.

## Figures and Tables

**Table 1:** The corresponding parameters of black phosphorus (BP).

Items	Crystal System	Space Group	Lattice Parameter (Å)		Bond Length (Å)		Interlayer Spacing (Å)	
			Armchair Direction	Zigzag Direction	P1–P2	P2–P3	Nearest Atomic	Layer-to-Layer Distance
This work	Orthorhombic	CMCA	4.55	3.28	2.20	2.24	3.10	5.24
Other work	-	-	4.58 [14] 4.54 [26] 4.54 [27]	3.32 [14] 3.28 [26] 3.31 [27]	-	-	3.20 [14]	-
Experimental value	-	-	4.37 [28]	3.31 [28]	-	-	-	-

**Table 2:** Data of BP nanotubes with different bending directions and curvature radii. The energy per atom of planar monolayer BP (*E*_a_) is −179.66 eV with a band gap of 0.763 eV.

Modes	r (Å)	Strain	Atom Number	*E*/Atom (eV)	*E*_b_ (eV/atom)	*E*_b_ (KJ/mol)	*E*_f_ (eV/atom)	*E*_f_ (KJ/mol)	Band Gap (eV)
aPNT	5.2	20.2%	36	−179.60	0.060	5.73	5.22	502.13	0.331
aPNT	7.3	14.4%	48	−179.62	0.034	3.30	5.24	504.05	0.492
aPNT	10.06	10.5%	64	−179.64	0.020	1.94	5.26	505.97	0.641
aPNT	11.5	9.1%	72	−179.65	0.016	1.55	5.27	506.93	0.678
aPNT	12.85	8.2%	80	−179.66	0.014	1.29	5.28	507.89	0.720
zPNT	7.3	14.4%	64	−179.49	0.163	15.66	5.11	491.55	0.291
zPNT	11.5	9.1%	96	−179.56	0.101	9.69	5.18	498.28	0.567

**Table 3:** The lattice parameters of armchair phosphorene nanotubes (aPNTs) and zigzag phosphorene nanotubes (zPNTs) with radii of 7.3 Å compared to planar monolayer BP.

Bonds	Planar	Bond Population	aPNT	Deviation (%)	Bond Population	zPNT	Deviation (%)	Bond Population
P1–P2	2.20	0.84	2.23	1.3	0.87	2.41	9.2	0.32
P2–P3	2.24	0.40	2.25	0.4	0.34	2.20	−2.1	0.43
P3–P4	2.20	0.84	2.19	−0.7	1.05	2.15	−2.2	0.36
P1–P2–P3	103.86	-	100.09	−3.6	-	96.93	−6.7	-
P3–P4–P5	103.86	-	109.13	5.1	-	107.57	3.6	-
